# Robotic-assisted Unicompartmental knee Arthroplasty optimizes joint line restitution better than conventional surgery

**DOI:** 10.1186/s40634-020-00309-8

**Published:** 2020-11-30

**Authors:** Roberto Negrín, Jaime Duboy, Nicolás O. Reyes, Maximiliano Barahona, Magaly Iñiguez, Carlos Infante, José Antonio Cordero, Vicente Sepulveda, Gonzalo Ferrer

**Affiliations:** 1 Department of Orthopedics and Traumatology, Clínica Las Condes, Estoril 450, Las Condes, Santiago, Chile; 2 Department of Orthopaedic Surgery, Hospital Clínico de la Universidad de Chile, Santiago, Chile

**Keywords:** Unicompartmental arthroplasty. Robotic-assisted surgery. Joint line

## Abstract

**Purpose:**

To compare joint line restoration after unicompartmental knee arthroplasty (UKA) between conventional and robotic-assisted surgery. Previous studies have shown that joint line distalization can lead to higher failure rates. The hypothesis was that robotic-assisted UKA is associated with less femoral component distalization and a precise tibial cut, which allows a more anatomical restitution of the knee joint line.

**Methods:**

Retrospective cohort study of patients undergoing medial or lateral UKA between May 2018 and March 2020. Preoperative and postoperative radiologic assessment of the joint line was performed by two observers, using three different methods, one for tibial slope and one for tibial resection. Robotic assisted UKA and conventional UKA groups were compared.

**Results:**

Sixty UKA were included, of which 48 (77.42%) were medial. Robotic-assisted UKA were 40 (64.52%) and 22(35.48%) were conventional

The distalization of the femoral component was higher in the conventional group despite the method of measurement used In both Weber methods, the difference was statistically different: Conventional 2.3 (0.9 to 5.6) v/s Robotic 1.5 (− 1.1 to 4.1) (p =0.0025*). A higher proportion of patients achieved a femoral component position ≤ two millimeters from the joint line using robotic-assisted UKA compared to the conventional technique .

No statistical difference between robotic-assisted and conventional UKA was found in tibial resection and slope.

**Conclusion:**

Robotic-assisted UKA shows a better rate of joint line restoration due to less femoral component distalization than conventional UKA. No difference was found in the amount of tibial resection between groups in this study.

**Level of evidence:**

III

## Introduction

Unicompartmental knee arthroplasty (UKAs) is a cost-effective treatment for severe femorotibial unicompartmental knee osteoarthritis. Remarkable advantages over total knee replacement (TKA) are a better restoration of native kinematics, less morbidity, and better recovery of joint function. However, it has higher revision rates [1, 2].

The main reason for lower survival is aseptic loosening, which has been related to the orientation of the components and the restoration of the joint line [3–5]. There are reports that associate higher failure rates when the joint line is not restored, despite if it is due to the distalization of the femoral component or an excessive tibial cut [4, 6].

Also, there is evidence that not restoring the joint line may lead to soft tissue imbalance, a higher incidence of anterior pain, and a limitation in post-operative knee flexion [7].

Therefore, to restore the joint line must be a crucial goal in UKA to reproduce the native knee biomechanics.

Robotic-assisted UKA has shown to improve the accuracy of the position of UKA components compared to conventional UKA [8–10]. Also, robotic-assisted UKA has shown to contribute to avoid excessive tibial resection [3, 11]. Hence, using robotic assistance may play a key role in restoring the joint line during UKA.

The purpose of this study is to compare the joint line restoration after UKA between conventional and robotic-assisted surgery. The hypothesis was that robotic-assisted UKA is associated with less femoral component distalization and a precise tibial cut, which allows an anatomical recovery of the knee joint line.

## Methods

### Patients

A retrospective cohort study of patients undergoing medial or lateral UKA was performed [8]. Consecutive patients who underwent UKA between May 2018 and March 2020 with the diagnosis of unicomparmental knee osetoarthrosis or unicondylar knee osteonecrosis that required surgery were included. There was no age limit for inclusion criteria. In all surgeries, the same model of UKA was used (Journey UNI, Smith & Nephew, Inc., Cordova, TN, USA). Surgeries were performed by different surgeons as a team. All after surgery, radiographs were analyzed and compared between patients that underwent robotic-assisted (Navio, Blue Belt Technologies, Plymouth, MN) and conventional UKA. Patients with other UKA models or those who did not have both pre- and post-surgery radiographs were excluded.

### Measurements

All x-ray measurements were made by two independent evaluators (JC, VS) with the Horos Project program (Nimble Co LLC, Purview, Annapolis, MD, USA.) Both observers have an equivalent level of expertise and received appropriate training to perform the measurements. Measurements were performed on both pre-surgery and 1 month post-surgery weightbearing anteroposterior (AP) and lateral knee radiographs.

**Fig. 1:**
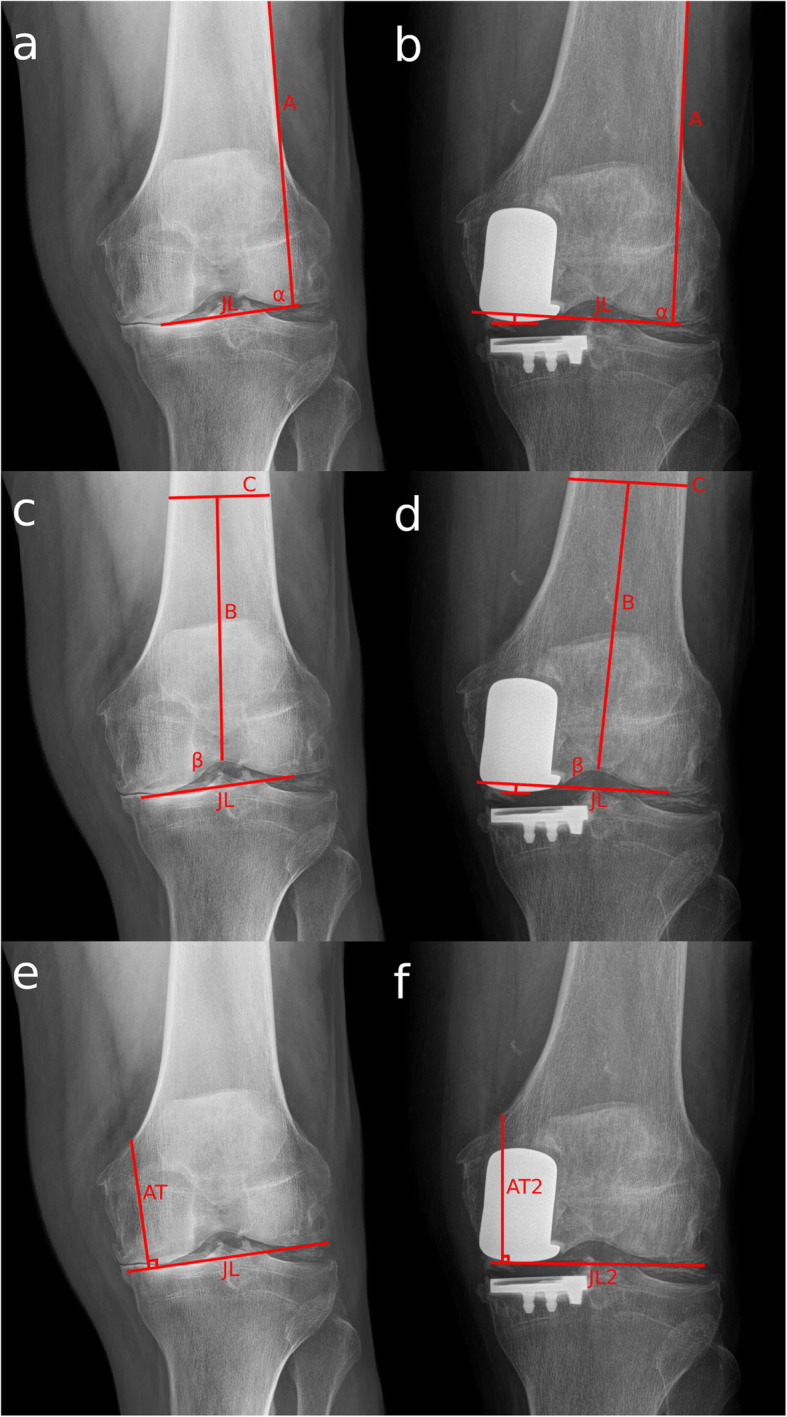
Anteroposterior Knee X-rays of a left knee showing the different joint line measure techniques. In the Weber 1 method, the angle (alpha) between a line that goes through the most distal point of both femoral condyles (JL) and the lateral femoral cortex (**a**) is obtained in the pre-operative x ray (**a**). Then (alpha) is reproduced in the post-operative x ray (**b**) with the lateral femoral cortex and the non-operative femoral condyle as reference. The variation in joint line is then measured. In the Weber 2 method the angle (beta) is obtained in the pre-operative x ray (**c**) between a line that goes through the most distal point of both femoral condyles (JL) and the line B that goes from the most proximal point of the intercondylar notch and the center of a line 10 cm proximal to it (**c**). Then the angle is reproduced in the post-operative x ray (**d**) with line B and the non-operative condyle as reference. The variation in joint line is then measured. In the Iacono method, the distance (AT) between the most proximal point of the Adductor Tubercle perpendicular to a line that goes through the most distal point of both femoral condyles (JL) (**e**) is compared to the same distance in the post-operative x ray (**f**). The difference between both is the variation in joint line

The restitution of the height of the joint line was assessed with the methods described by Weber and Iacono [7, 12]. Two angles were described in the Weber method [7]. The first angle was defined by a line that joins the most distal points of both femoral condyles and a second line tangent to the lateral cortex (method 1) (Fig. 1a,b).

In the second angle, the same tangent to the distal condyles was used, and a line was drawn from the highest point of the notch to a central point on the femoral diaphysis 10 cm. from the first point (method 2) (Fig. 1c,d). Both angles were reproduced on the post-surgery radiograph, using as reference the lateral femoral cortical, the anatomic femoral axis, and the non-operated condyle. Then, the height between the printed line and the center of the UKA implant was defined as the amount of distalization of the knee joint line after UKA.

A third method for the joint line distalization was used [12], which consisted of measuring the height of the joint line from the adductor tubercle perpendicular to a line that passed through the most distal point of both femoral condyles. The difference between the pre- and post-operative measurements resulted in the variation in the height of the joint line after UKA (Fig. 1e,f).

**Fig. 2:**
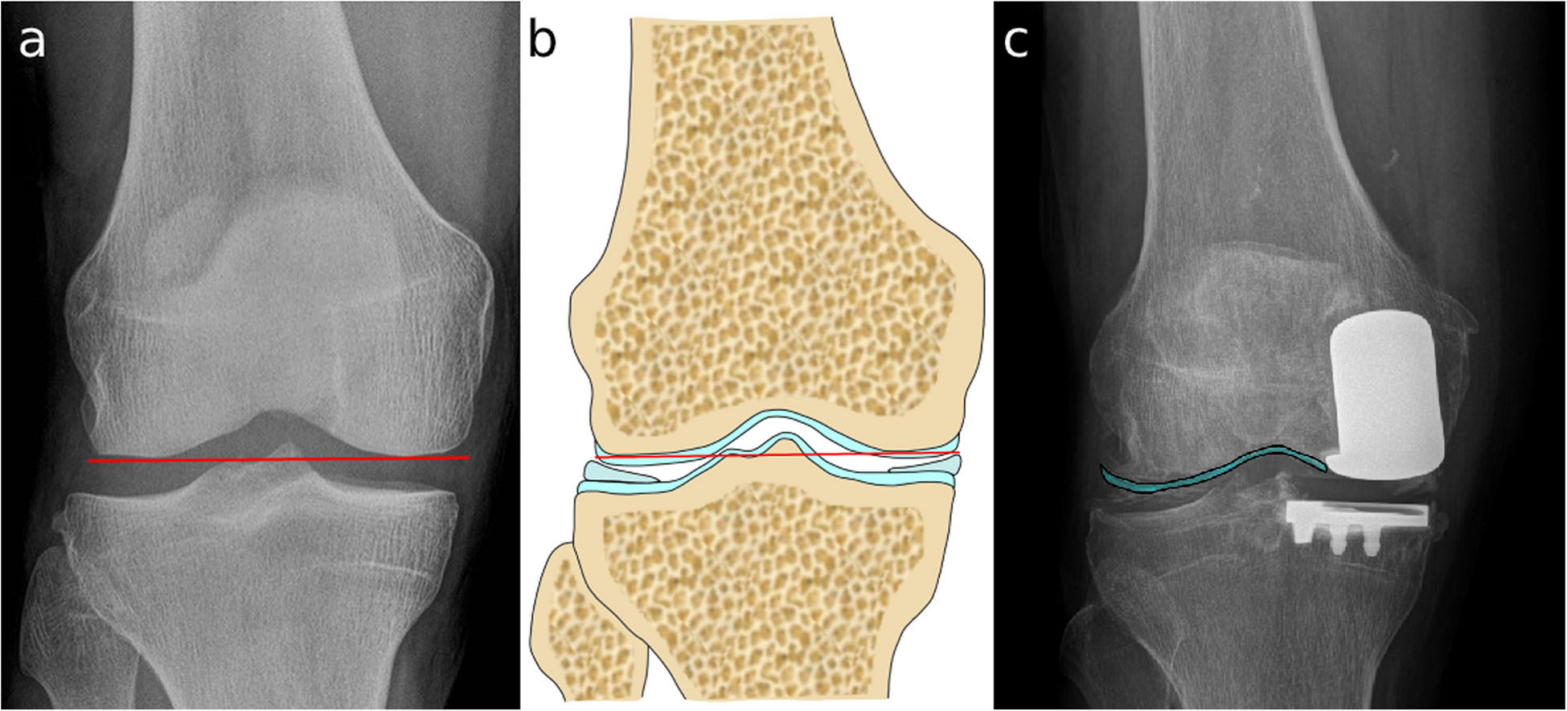
Joint line and cartilage (**a**) Radiological joint line measured in a native knee. **b** Shows the radiological joint line in the subchondral bone. The femoral cartilage is 2 mm thick. **c** Implant positioned in reference to the cartilage and not the subchondral bone

In all three measurements, a successful joint line restoration was defined when the femoral component was up to two millimeters deep to the native joint line. The two millimeters mark was used as anatomical and radiological studies have shown that the thickness of the hyaline cartilage in the femoral condyles is up to two millimeters [13, 14]. It should be noticed that radiographs do not show the cartilage itself, so up to two millimeters of distalization of the femoral component is desirable. (Fig. 2).

The pre- and post-operative tibial slope was evaluated with the method described by Dejour [15].

**Fig. 3:**
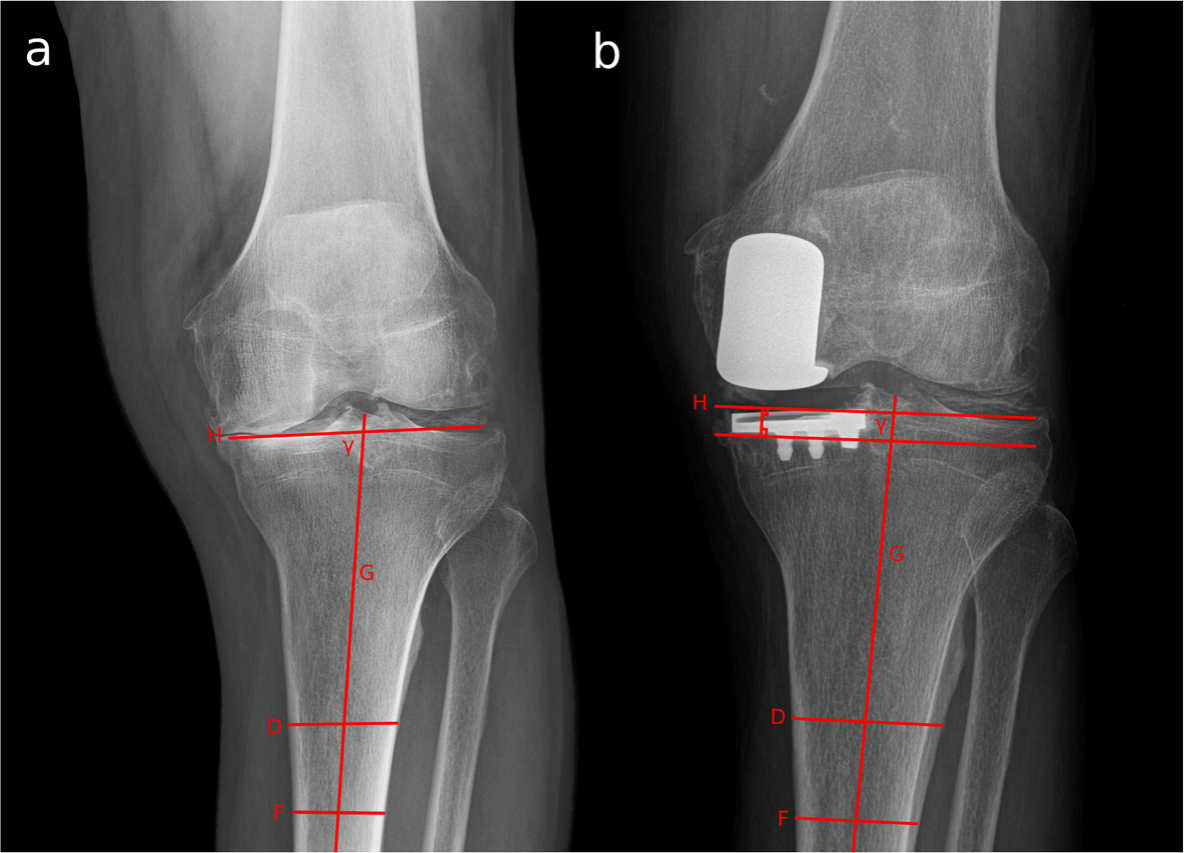
Anteroposterior Knee X-rays of a left knee showing the tibial resection measure method. The angle (gamma) is obtained in the pre-operative x ray (**a**) between a line (H) that goes through both tibial plateaus and a line that represents the tibial anatomical axis (G), obtained by the center of two lines (D and F) at 7 and 10 cm distal to the joint line. Then the angle is reproduced in the post-operative x ray (**b**) with the tibial anatomical axis (G) and the non-operative tibial plateau. The distance from the line H to the base of the implant represents the tibial resection

Finally, tibial resection was measured using a novel method. First, in an AP radiograph of the knee, the anatomical proximal tibia angle (APTA) angle was measured. In the case of a lateral UKA, the medial APTA was measured, and if it was a medial UKA, the lateral APTA was measured. In the post-surgery AP radiograph, the angle was reproduced, using the healthy tibial plateau. Then the distance of the reproduced APTA to the bottom of the femoral component was measured using a perpendicular line and was interpreted as the amount of tibial resection. (Fig. 3).

### Statistics

The interobserver agreement was evaluated using the concordance correlation coefficient (CCC) described by Lin [16] and the Bland and Altman limits of agreements [17]. A good concordance was defined if CCC was higher than 0.80, or a probability above 0.10 was reached using the Bradley Blackwood F test for the Bland and Altman limits of agreements [18]. An excellent concordance was considered if both parameters were achieved. The average of the measurement of both observers was used for analyses.

Frequency, proportions, median, range, and interquartile range were used to describe the study sample. Robotic and conventional UKA were compared using the median non-parametric test for continuous variables distalization of the femoral component, tibial resection, and slope. Fisher’s exact test was used to compare categorical variables: the proportion of medial UKA, the ratio of the right knee, and the size of the tibial insert. Power was estimated for a two sample comparison of proportions using the normal aproximation to the binomial distribution.

Ordinal regression was estimated to predict the size of the tibial insert. First, a univariate estimation was performed and then using the significant independent variables, multivariate ordinal regression was estimated. A probability > 0.15 in the Brant test was used to test that the parallel regression assumption was not violated. Odds ratio (OR) was calculated, and the 95% confidence intervals were constructed, and a significance of 0.05 was used. Data was processed using Stata version 11.2 (StataCorp LP, College Station, Texas, USA).

## Results

A total of 62 UKA were included, of which 48 (77.42%) were medial, and 32 (52.46%) were right-sided. Robotic-assisted UKA were 40 (64.52%), no statistical difference was found in the proportion of medial UKA, but there were significantly more left-sided in the robotic group. The average age for the robotic group was 67.7 and 66.45 for the conventional group (Table 1).

**Table 1:** The comparison between conventional and robotic-assisted UKA is summarized. No difference was found in the proportion of medial UKA, but a higher percentage of right knee underwent conventional UKA

	**Conventional**	**Robotic**	***p***
**N**	22 (35.48%)	40 (64.52%)	
**Medial**	16 (72.73%)	32 (80.00%)	0.539
**Right**	17 (77.27%)	15 (37.50%)	0.004

The two Weber methods achieved excellent concordance. The slope measurement made a good concordance according to CCC, and the Iacono method and tibial resection, according to Brand-Altman (Table 2).

**Table 2:** The assessment of concordance is summarized. The 95% confidence interval is shown in square brackets

	CCC^a^	**Brand-Altman**	**F test**^b^
**Weber 1**	0.88 [0.82–0.94]	0.065 [− 1.15–1.28]	0.32
**Weber 2**	0.90 [0.86–0.95]	0.059 [−1.09–1.28]	0.64
**Iacono**	0.70 [0.57–0.83]	−0.16 [−2.01–1.97]	0.45
**Slope before surgery**	0.83 [0.75–0.90]	−0.025 [−2.78–2.72]	0.001
**Slope after surgery**	0.94 [0.91–0.97]	−0.157 [−2.23–1.91]	0.02
**Tibial resection**	0.75 [0.64–0.86]	0.097 [−1.86–2.06]	0.22

^a^Concordance correlation coefficient

^b^Bradley – Blackwood F test

**Fig. 4:**
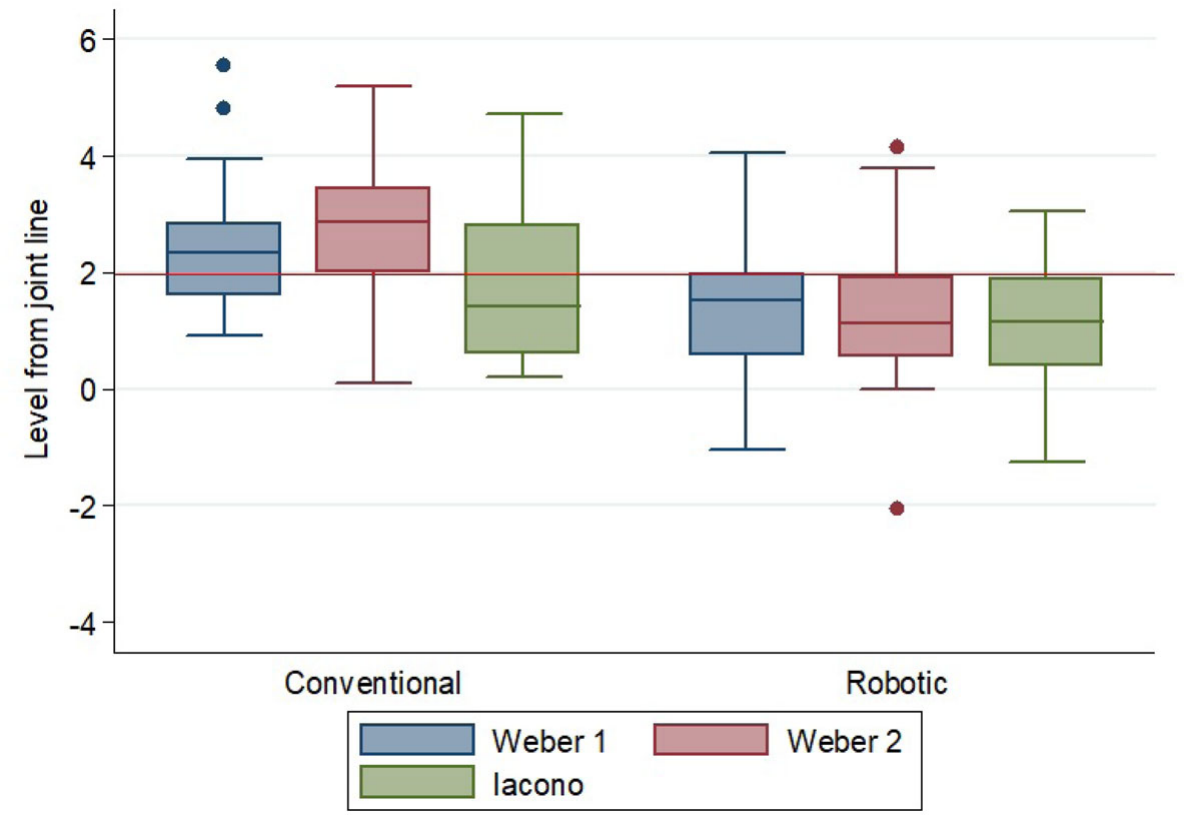
The distribution of the position of the femoral component compared to the femoral joint line. Three methods were used to differentiate between the robotic-assisted and conventional groups

The distalization of the femoral component was higher in the conventional group despite the method of measurement used (Fig. 4). In both Weber methods, the difference was statistically different (Table 3).

**Table 3:** Radiological measurements were compared between groups, and a significant difference was found in both Weber methods. The range is summarized between round brackets and the interquartile range between square brackets

	**Conventional**	**Robotic**	***p***
**Weber 1**	2.3 (0.9 to 5.6) [1.6 to 2.9]	1.5 (− 1.1 to 4.1) [0.6 to 2.0]	0.0025*
**Weber 2**	2.9 (0.1 to 5.2) [2.0 to 3.5]	1.1 (−2 to 4.2) [0.6 to 1.9]	< 0.0000*
**lacono**	1.4 (0.2 to 4.7) [0.6 to 2.8]	1.2 (−1.3 to 3.1) [0.4 to 2.0]	0.1277*
**Slope** **Before surgery**	8.0 (4.6 to 12.5) [5.7 to 9.3]	8.2 (2.1 to 13.8) [6.5 to 9.9]	0.4754*
**Slope** **After** **Surgery**	5.7 (0.9 to 10.7) [4.8 to 8.9]	6.4 [0.6 to 14.7] (4.3 to 8.3)	0.9355*
**Slope differential**	−1.4 (− 7.9 to 6.15) [− 4.3 to 0.8]	−2.0 (− 7.4 to 4.6) [− 4.0 to 0.1]	0.8082*
**Tibial resection**	5.8 (3.5 to 8.9) [4.4 to 6.5]	6.2 (3.1 to 8.7) [5.3 to 7.1]	0.5237*
**Tibial insert**	8 (8 to 10) [8 to 9]	9 (8 to 11) [8 to 9]	0.3464**

*non-parametric median test

**Fisher exact test

**Fig. 5:**
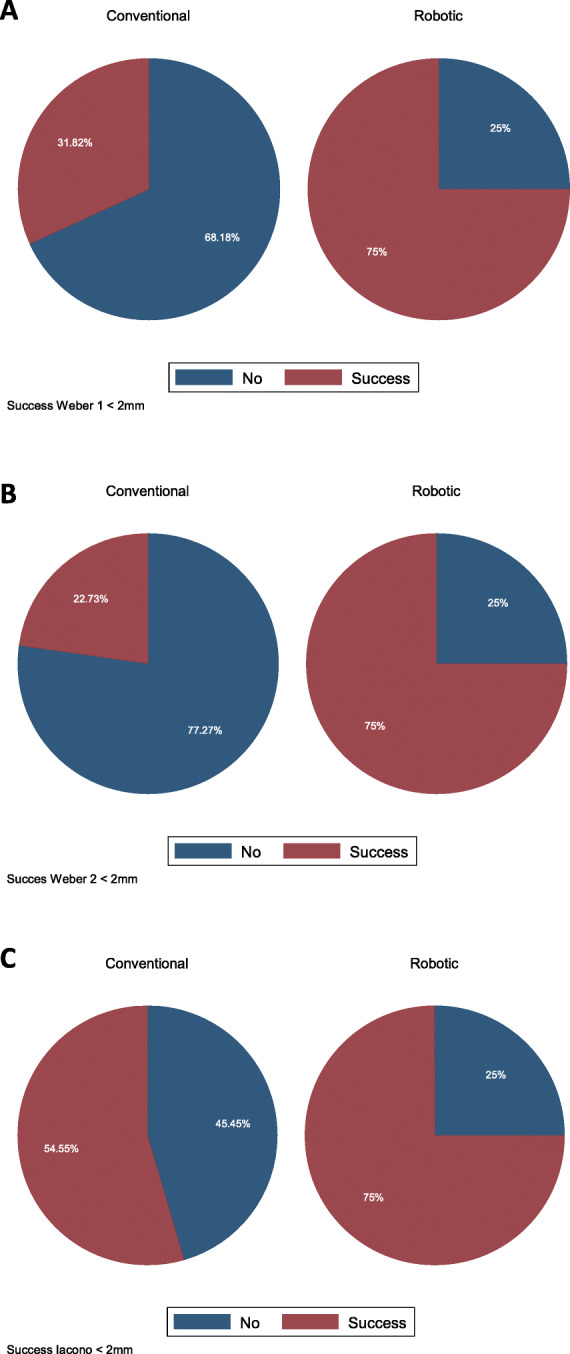
Graph pie showing the proportion of patient that achieve the position of the femoral component two millimeters or below to the femoral joint line by type of UKA according to Weber 1 method (**a**) Weber 2 method (**b**) and Iacono method (**c**)

A higher proportion of patients achieved a femoral component position below two millimeters from the joint line using robotic-assisted UKA compared to the conventional technique. (Table 4, Fig. 5).

**Table 4:** The proportion of patients that achieve the position of the femoral component two millimeters or below to the femoral joint line by type of UKA is shown, according to the three methods

	**Conventional**	**Robotic**	**Fisher exact test (p**)
**Weber 1**	7 (31.82%)	30 (75.00%)	0.001
**Weber 2**	5 (22.73%)	30 (75.00%)	< 0.000
**Iacono**	12 (54.55%)	30 (75.00%)	0.16

No statistical difference between robotic-assisted and conventional UKA was found in tibial resection and slope measurements (Table 3). The median tibial insert size was 9 (interquartile range: 8 to 9) in the robotic UKA and 8 (interquartile range: 8 to 9) in the conventional group, not reaching statistical difference.

The ordinal regression estimates that in this cohort, the femoral cut measured by any of the Weber methods was the main variable to predict the size of the tibial insert. The Weber 1 method reached an OR of 0.55 (0.35–0.86) and the Weber 2 an OR of 0.58 (0.38–0.88) for a smaller tibial insert. Robotic-assisted UKA trend to greater tibial inserts, consistent with a higher proportion of less distalized femoral components, but did not reach significance in the multivariate ordinal regression (OR, 2.24; 95% confidence interval 0.84–7.19). Also, tibial resection did not reach statistical difference (OR, 1.19; 95% confidence interval 0.83–1.72).

## Discussion

Our study shows that robotic-assisted UKA allows for better restoration of the knee joint line when it is compared to conventional UKA. Though several studies showed that robotic-assisted surgery improves the precision of the position of the implants and decreases the outliers [8–10], there is little literature that approaches the restitution of the joint line.

We used the Journey UNI implants in our study in which the femoral component has a thickness of 5.5 mm. for components size 1 and 2 and of 6.5 mm. for sizes 3 to 7. In the conventional technique, the distalization of the joint line is inevitable, due that the sum of the thickness of the cartilage plus the resection of the subchondral bone is less than the thickness of the femoral implant and the conventional technique only allows two options for the width of the femoral cut [19]. Meanwhile, in the robotic-assisted technique, the femoral cut width can be customized according to the desired position of the femoral component.

In the robotic-assisted group, a significantly better rate of success was achieved with both Weber methods compared to the conventional group, as was the case in the Herry et al. study [19]. Using the Iacono method, a better rate of success was observed; nevertheless, it did not achieve statistical significance [12].

Lo Presti et al. [4] showed in their results that in the group that presented early failures, the joint line was distalized 4.3 mm. on average. Our robotic-assisted group presented in 75% a distalization of less than 2 mm.

When we measure the proximal tibial resection with our method, there is no statistically significant difference found between the robotic-assisted and conventional groups. This finding differs from literature, that shows a direct relationship between joint line distalization and tibial resection [6].

Since no difference in the amount of tibial resection was found between groups and that the robotic-assisted technique allows the surgeon to achieve a better soft tissue balance, we think that the difference is absorbed by a higher soft tissue tension in the conventional group. The soft tissue balance is ensured by the software included in the robotic assistance, evaluating the pressure in the compartment through the entire range of motion. On the other hand, in the conventional UKA, in which we find a greater distalization in this study, the tension must be increased in the compartment, unless a smaller insert size balances it sufficiently.

The size of the tibial inserts shows no difference between groups. This finding differs from the report of Ponzio and Lonner, as they found that in the robotic-assisted surgery, a less aggressive tibial resection was performed [20]. This was inferred using the tibial insert size, and no measurement of the amount of femoral joint line distalization was performed [20]. Moreover, in our study, the ordinal regression showed that the femoral cut directly influences the tibial insert size, as for each millimeter that the joint line is distalized there is an 82% probability of using a smaller insert. Although not significant, there is a trend in robotic-assisted surgery to use bigger inserts in association to a less distalized femoral component. In conventional surgery a more distalized femoral component has to be compensated with a smaller tibial insert for an adecuate soft tissue balance.

Iñiguez et al. [10] showed that robotic-assisted surgery improves the selection of the size of the femoral component, being this factor intimately related to the distalization of the joint line. To use the adequate femoral component size for the patients´ condyle is paramount to restore the anatomic femoral joint line.

A median tibial cut of 6 mm was found in this study, which is considered appropriate, as it is related to fewer cases of subsidence, less insert overload, and to lower post-operatory pain [21]. Furthermore, a more precise cut has an 80% probability of requiring a primary TKA if a UKA revision is needed [22].

No difference was found between groups regarding tibial slope. During UKA, restoring the native slope is desirable. Navio allows the surgeon to plan the slope during the tibial cut making this tibial slope cut more reproducible. In our cadaveric pilot study, we did not obtain differences in the tibial slope either, when comparing robotic-assisted to conventional surgery [10].

A strength of our study includes the use of three validated joint line measurements. Also, this method shows good to excellent interobserver agreement, which allows us to use the mean of the measurements reported by each observer.

A limitation of this study is that the joint lines were assessed using radiographs, and therefore, there may be variations due to limb rotation or inclination. However, the Weber method shows that it has a tolerance of 10 degrees of rotation to be effective [9]. Probably the best way to measure this would be with a CT, but we consider that over-exposing our patients to the radiation that this implies is not justified [20]. Another limitation is that the alignment of the limb was not measured, which is related to the joint line, as Kuwashima et al. [23] showed in their study. Another limitation is that the sample size may have been underpowered. Also, one of the more relevant limitations was that the method used to measure the tibial resection was not validated; nevertheless, due to the good agreement achieved by the two evaluators, we think that it is reproducible. Even more, the resection was consistent with the size of the tibial insert. Finally, other limitation was that surgeries were performed by more than one surgeon, altough, they were performed as a team.

Due to the results obtained in this study, it will be essential to follow this cohort to analyze differences in functional outcomes, aseptic loosening, and survival. Future studies must aim to determine if this radiological measurement difference means better clinical results in UKA, which will mean a greater need for robotic assistance in this surgery.

## Conclusions

Robotic-assisted UKA shows a better rate of joint line restoration due to less femoral component distalization than conventional UKA. No difference was found in the amount of tibial resection between groups in this study.
